# Supplementary material for: Lysophospholipids as Predictive Markers of ST-Elevation Myocardial Infarction (STEMI) and Non-ST-Elevation Myocardial Infarction (NSTEMI)
Source: Metabolites. 2020 Dec 31;11(1):25. doi: 10.3390/metabo11010025 (PMC7823877; doi:10.3390/metabo11010025)
Supplement: Supplementary file 1 [file metabolites-11-00025-s001.zip › metabolites-1033293-supplementary-update/Supplementary material S1.docx]

**Supplementary material**

**Metabolomic analyses of plasma samples**

Prior to GC-TOF/MS metabolomics analysis, a further two-step derivatization procedure was carried out to increase metabolites volatility (silylated) and reduce number of tautomeric form of each carbohydrate (methoxymated) (A et al. 2005). The derivatized samples (1 μL) were analyzed on an Agilent 6890 gas chromatograph equipped with a 10 m × 0.18 mm i.d. fused silica capillary column with a chemically bonded 0.18-µm DB 5-MS stationary phase (J&W Scientific, Folsom, CA). The injector temperature was 270 °C. The column temperature was held at 70 °C for 2 min, increased by 40 °C min^-1^ to 320 °C, and held there for 1 min. The column effluent was introduced into the ion source of a Pegasus III time-of-flight mass spectrometer, GC-TOF/MS (Leco, St. Joseph, MI, USA). The transfer line and the ion source temperatures were 250 and 200 °C, respectively. Ions were generated by a 70-eV electron beam at an ionization current of 2.0mA. The spectra were recorded in the mass range 50–800 m/z at a rate of 30 spectra s^-1^.

Prior to LC-TOF/MS analyses, the samples were re-suspended in 10 + 10 μL of methanol and water for metabolomic analysis. Aliquots of muscle extracts (2 μL) were injected onto a Waters Acquity UPLC HSS T3 C_18_ column (2.1 × 50 mm, 1.8 μm, Waters, Milford, MA, USA) in combination with a 2.1 mm x 5 mm, 1.8 µm VanGuard precolumn (Waters Corporation, Milford, MA, USA) held at 40 °C. The chromatographic separations of metabolites were carried out using a gradient solvent system consisted of water with 0.1 % formic acid (A) and acetonitrile/isopropanol (75/25, v/v) with 0.1 % formic acid (B) at a flow rate of 0.5 mL min^-1^. The gradient started at 0.1 % B increasing linearly to 10 % B in 2 min and increased to 99 % B over 5 min which held for 2 min; the proportion of B was decreased to 0.1 % for 0.3 min and was further decreased to 0 % with the flow-rate increased to 0.8 mL min^-1^ for 0.5 min which held for 0.9 min; the initial conditions were restored in 0.1 min before the next injection.

The detection of separated metabolites was performed using the Agilent 6550 Q-TOF mass spectrometer equipped with a jet stream electrospray ionization (ESI) source, operating in both positive and negative ion modes. A reference interface was connected for accurate mass measurements and reproducibility. Full scan MS spectra were collected in a centroid mode over the mass range 70-1700 m/z with an acquisition rate of 4 spectra s^-1^. The capillary voltage was set at +4 kV and –4 kV with nozzle voltages of +300 V and –300V for positive and negative ion modes, respectively. Other MS parameters were applied as follows: gas temperature was set at 150 °C, drying gas flow was 16 L min^-1^, and the pressure of nebulizer gas was 35 psig. The sheath gas flow was kept at 11 L min^-1^with a temperature of 350 °C. The voltages of the fragmentor, skimmer, and octopole RF peak voltage were 380 V, 45 V and 750 V, respectively. The LC-TOF/MS data acquisition was performed in global profiling mode including the initial MS/MS scanning for the further targeted and untargeted data analysis. Additional targeted and untargeted MS/MS analyses of quality control samples were performed for targeting metabolites included in the in-house database and for elucidating the structures of untargeted metabolites, respectively. Data were acquired with MassHunter Acquisition Software B.05.01.

***Data processing***

For the GC-data, an in-house MATLAB script was used for the extraction of putative metabolites by matching the mass spectra and retention indices to in-house mass spectral library at the Swedish Metabolomics Centre and the publicly available Max Planck Institute library in Golm. The processing of LC-TOF/MS data and extraction of putative metabolites and lipids were performed by MassHunter Profinder version B.08.00 in combination with Qualitative Analysis software version B.07.00, PCDL manager version B.07.00 and Mass Profiler Professional™ 13.0 (all from Agilent Technologies Inc., Santa Clara, CA, USA). Annotation of putative metabolites were done by matching the retention time and mass spectra (MS and MS-MS spectra) against the in-house metabolite and lipid library. By using metabolic profiling mode, numerous of unknown putative metabolites and lipids, not available in current databases, could be quantified and included in sample comparison modelling.

All detected and annotated metabolites are listed in table S1.
